# Urinary endotrophin predicts disease progression in patients with chronic kidney disease

**DOI:** 10.1038/s41598-017-17470-3

**Published:** 2017-12-11

**Authors:** Daniel Guldager Kring Rasmussen, Anthony Fenton, Mark Jesky, Charles Ferro, Peter Boor, Martin Tepel, Morten Asser Karsdal, Federica Genovese, Paul Cockwell

**Affiliations:** 1 Nordic Bioscience, Herlev, Denmark; 2 University of Southern Denmark, Institute of Molecular Medicine, Cardiovascular and Renal Research, Odense, Denmark; 3 Department of Renal Medicine, Queen Elizabeth Hospital, Birmingham, UK; 4 College of Medical and Dental Sciences, University of Birmingham, Birmingham, B15 2TT UK; 5 Division of Nephrology, RWTH University of Aachen, Aachen, Germany; 6 Institute of Pathology, RWTH University of Aachen, Aachen, Germany; 7 Department of Nephrology, Odense University Hospital, Odense, Denmark

## Abstract

Renal fibrosis is the central pathogenic process in progression of chronic kidney disease (CKD). Collagen type VI (COL VI) is upregulated in renal fibrosis. Endotrophin is released from COL VI and promotes pleiotropic pro-fibrotic effects. Kidney disease severity varies considerably and accurate information regarding CKD progression may improve clinical decisions. We tested the hypothesis that urinary endotrophin derived during COL VI deposition in fibrotic human kidneys is a marker for progression of CKD in the Renal Impairment in Secondary Care (RIISC) cohort, a prospective observational study of 499 CKD patients. Endotrophin localised to areas of increased COL VI deposition in fibrotic kidneys but was not present in histologically normal kidneys. The third and fourth quartiles of urinary endotrophin:creatinine ratio (ECR) were independently associated with one-year disease progression after adjustment for traditional risk factors (OR (95%CI) 3.68 (1.06–12.83) and 8.65 (2.46–30.49), respectively). Addition of ECR quartiles to the model for disease progression increased prediction as seen by an increase in category-free net reclassification improvement (0.45, 95% CI 0.16–0.74, p = 0.002) and integrated discrimination improvement (0.04, 95% CI 0.02–0.06, p < 0.001). ECR was associated with development of end-stage renal disease (ESRD). It is concluded that ECR predicts disease progression of CKD patients.

## Introduction

Chronic kidney disease (CKD) is associated with end-stage renal disease (ESRD) and increased risk of mortality^1,2^. Progressive fibrosis is the major pathophysiological process in CKD and collagens are the main structural components of the fibrotic tissue^1,3,4^. As collagen formation and fibrosis progression are closely linked, assessment of active collagen formation may identify CKD patients at high risk of progression, including those who have the greatest potential for targeted anti-fibrotic therapies^5,6^.

COL VI is an important structural collagen positioned at the interface between the interstitial matrix and the glomerular basement membrane. It forms an intricate meshwork of microfilaments with a physiological role in maintaining structure and function, controlling the organization of the matrix and cell orientation^7^. Increased deposition in the kidneys has been reported in animal models of kidney disease and in CKD in humans^8,9^.

Recent seminal studies have shown that collagens can also interact with both their immediate surroundings and distant sites through the release of fragments with stimulating and signalling activity, termed matrikines^6,10^. During production of COL VI, the pro-peptide of the alpha-3 chain is released, containing endotrophin^11,12^. Due to sequence overlap in endotrophin and the pro-peptide of COL VI, endotrophin may be a potential surrogate biomarker for COL VI formation. Endotrophin also promotes biological effects, such as acting as a chemoattractant on macrophages, increasing TGFβ signalling, and epithelial-mesenchymal transition^13^. Additional systemic effects include adipose tissue fibrosis and metabolic dysfunction^13^. We have recently shown that increased serum endotrophin is independently associated with mortality in CKD patients^14^ and elevated in renal transplant recipients with chronic rejection^15^. However, the role of urinary endotrophin in the pathophysiology of CKD has not been elucidated. We tested the hypothesis that endotrophin is produced in fibrotic human kidneys and that urinary endotrophin is a marker for progression of CKD in a prospective observational study of 499 CKD patients.

To address this, we analysed the localization of endotrophin and COL VI *in situ* and studied the association of urinary endotrophin:creatinine ratio (ECR) with one-year disease progression and development of ESRD in a large prospective study of patients with high-risk CKD.

## Results

### Presence of endotrophin in fibrotic kidney

**Figure 1:**
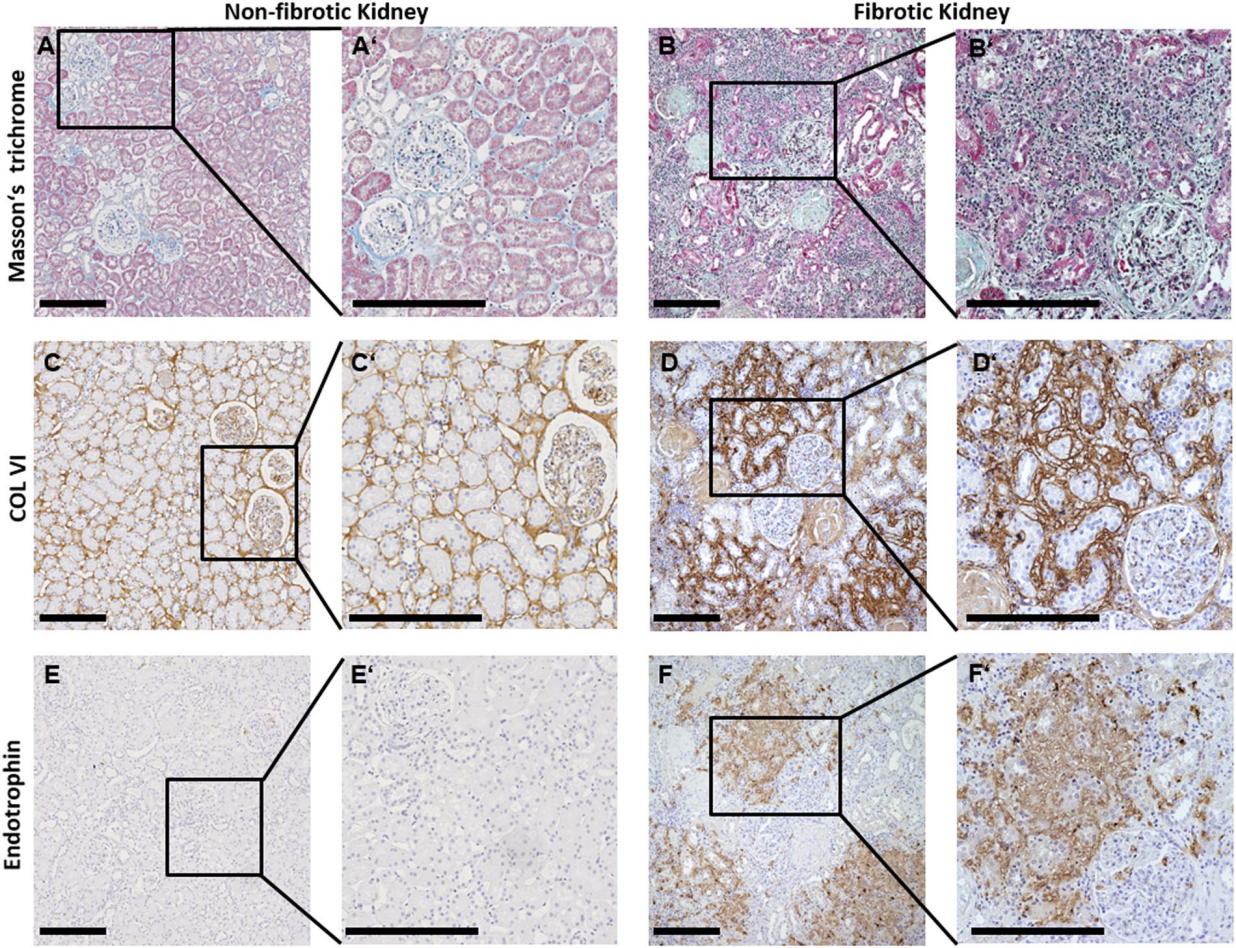
Histological and immunohistological assessment of tissue sections from control and fibrotic kidneys. The left (A, C, E and A’, C’, E’) and right panel (B, D, F and B’, D’, F’) show representative sections from two paraffin-embedded biopsies of a non-fibrotic kidney and fibrotic kidney, respectively. (**A**,**B**) Whereas Masson’s trichrome stain led to a very mild and only focal positivity in the control kidneys, a more intense staining, was observed in the fibrotic kidney. (**C**) Immunohistochemistry of the non-fibrotic control kidneys using anti-COL VI antibody (αCol6) revealed some staining in the interstitium and surrounding larger blood vessels. (**D**) The fibrotic kidneys showed noticeable, αCol6 staining in the fibrotic areas, indicating a prominent upregulation of COL VI in fibrosis. (**E**) Immunohistochemistry of the non-fibrotic kidneys using the anti-Endotrophin antibody (PRO-C6), did not reveal any staining. (**F**) The fibrotic kidney section showed a clear Endotrophin signal within the fibrotic foci, particularly those with high COL VI content (1D’ and F’). The subfigures labelled with a mark (e.g. A’) are magnifications of the areas outlined with a square. In the fibrotic kidney, the square outlines the same area in subfigures (**B**, **D** and **F**). The scale bars are 250 µm.

Masson’s trichrome staining in the non-fibrotic kidneys revealed only a focal and mild positivity (Fig. 1A), whereas in the fibrotic kidneys intense collagen staining was observed (Fig. 1B). Staining of COL VI showed low expression in the interstitium and surrounding larger blood vessels in the non-fibrotic kidney (Fig. 1C). In accordance with previous studies^8,9^, the fibrotic kidneys revealed a noticeable staining of COL VI indicating a prominent deposition in fibrotic areas (Fig. 1D). Staining for endotrophin showed no signal in the non-fibrotic kidneys suggesting very low COL VI turnover (Fig. 1E). In fibrotic kidneys, a prominent staining for endotrophin was observed, in areas that co-localized with high COL VI staining (Fig. 1F).

### Baseline characteristics of study subjects

The cohort was 61.5% male, median age was 64 years (IQR 50–76), and ethnicity was 72.2% white, 18.0% south-Asian, 8.8% Black, and 1.0% other. The primary renal diagnosis was ischaemia/hypertension in 26.1%, diabetic kidney disease in 9.6%, glomerulonephritis in 16.6%, polycystic kidney disease in 5.8%, and of other or uncertain aetiology in 41.9%. Median (IQR) estimated glomerular filtration rate (eGFR) was 27 (20–35) mL/min/1.73 m^2^, median (IQR) for urinary albumin:creatinine excretion ratio (ACR) was 32 (6–128) mg/mmol, and median (IQR) ECR was 0.7 (0.3–2.2) ng/µmol.

Baseline variables for the study participants stratified by ECR quartile, are presented in Table 1. Subjects in the higher quartiles were older, had higher probability of having diabetes as the primary renal diagnosis and as comorbidity, had lower probability of having glomerulonephritis, and in general had a higher level of comorbidity (age-adjusted Charlson’s Comorbidity Index (CCI) score above 5). Also, in subjects of the highest quartiles, a higher systolic blood pressure, mean arterial blood pressure (MAP), pulse pressure (PP), serum creatinine, cystatin C, ACR and lower kidney function was observed. None of the other variables were significantly different between the groups.

**Table 1:** Clinical characteristics of study subjects stratified by quartiles of ECR.

**Patient number ECR (IQR)**	**All**	**ECR Quartile**				**P value**
	**(n = 499) (0.3–2.2)**	**1 (n = 125) (0.1–0.2)**	**2 (n = 126) (0.3–0.5)**	**3 (n = 123) (0.9–1.7)**	**4 (n = 125) (3.6–8.8)**	
Age (years)	64 (50–76)	60 (49–72)	65 (50–78)	66 (52–78)	66 (54–76)	**0.02**
Gender (male)	307 (61.5)	79 (63.2)	71 (56.3)	79 (64.2)	78 (62.4)	0.57
Ethnicity						
White	360 (72.2)	97 (77.6)	95 (75.4)	85 (69.1)	83 (66.4)	0.16
Black	44 (8.8)	13 (10.4)	11 (8.7)	13 (10.6)	7 (5.6)	0.48
South Asian	90 (18.0)	14 (11.2)	19 (15.8)	23 (18.7)	34 (27.2)	**0.008**
Other	5 (1.0)	1 (0.8)	1 (0.8)	2 (1.6)	1 (0.8)	0.89
Primary renal diagnosis						
Ischaemia/hypertension	130 (26.1)	30 (24.0)	35 (27.8)	31 (25.2)	34 (27.2)	0.9
Diabetes mellitus	48 (9.6)	11 (8.8)	4 (3.2)	11 (8.9)	22 (17.6)	**0.002**
Glomerulonephritis	83 (16.6)	34 (27.2)	23 (18.3)	18 (14.6)	8 (6.4)	**0.0002**
Polycystic kidney disease	29 (5.8)	6 (4.8)	10 (7.9)	10 (8.1)	3 (2.4)	0.16
Other/Unknown	209 (41.9)	44 (35.2)	54 (42.8)	53 (43.2)	58 (46.4)	0.33
Co-morbidities						
Malignancy	72 (14.4)	15 (12.0)	19 (12.7)	17 (13.8)	21 (16.8)	0.74
Diabetes mellitus	183 (36.7)	39 (31.2)	37 (29.4)	47 (38.2)	61 (48.8)	**0.006**
COPD	60 (12.0)	16 (12.8)	22 (17.5)	11 (8.9)	11 (8.8)	0.12
Cerebrovascular disease	54 (10.8)	15 (12.0)	13 (10.3)	11 (8.9)	15 (12.0)	0.84
Ischaemic heart disease	112 (22.4)	30 (24.0)	30 (23.8)	22 (17.9)	30 (24.0)	0.58
Peripheral vascular disease	51 (10.2)	12 (9.6)	11 (8.7)	16 (13.0)	12 (9.6)	0.69
Age-adjusted CCI (score ≥ 5)	278 (55.7)	52 (41.6)	73 (57.9)	72 (58.5)	81 (64.8)	**0.0004**
Smoking (Current)	67 (13.4)	21 (16.8)	20 (15.9)	15 (12.2)	11 (8.8)	0.31
Socioeconomic status	29.0 (16.5–45.1)	28.7 (16.3–44.1)	25.5 (15.3–45.1)	33 (19.3–44.6)	27 (17–47)	0.66
BMI (kg/m^2^)	29 (25–33)	30 (26–33)	29 (25–32)	28 (25–33)	28 (24–35)	0.53
Systolic BP (mmHg)	124 (114–139)	119 (109–129)	123 (113–140)	126 (116–142)	127 (116–148)	**<0.0001**
Diastolic BP (mmHg)	74 (67–83)	74 (66–82)	75 (67–81)	74 (67–84)	75 (68–83)	0.92
Mean arterial pressure (mmHg)	91 (84-99)	90 (83–96)	91 (84–98)	94 (84–101)	94 (86–100)	**0.02**
Pulse pressure (mmHg)	48 (37–63)	42 (34–53)	49 (36–66)	51 (39–65)	51 (40–68)	**<0.0001**
Serum creatinine (μmol/L)	203 (161–259)	158 (137–197)	189 (149-225)	216 (181–260)	270 (222-345)	<0.0001
Cystatin C (mg/L)	2.5 (2.0–3.1)	1.9 (1.6–2.3)	2.3 (1.8-2.8)	2.5 (2.0–3.0)	3.1 (2.6–3.7)	<0.0001
eGFR (mL/min/1.73m^**2**^ **)**	27 (20–35)	36 (28–46)	28 (23–38)	25 (20–29)	18 (14–24)	**<0.0001**
ACR (mg/mmol)	32 (6–128)	10 (2–76)	23 (6–129)	47 (9–163)	77 (15–184)	**<0.0001**
CRP (mg/L)	3.1 (1.5–6.8)	2.8 (1.1–6.5)	3.6 (1.5–8.0)	2.7 (1.5–5.0)	3.9 (1.9–8.1)	0.18

Categorical variables are expressed as number (%), and continuous variables as median (IQR). Below the quartile headers the IQR for ECR has been inserted (ng/µmol creatinine). COPD, chronic obstructive pulmonary disease; CCI, Charlson’s comorbidity index; BMI, body mass index; BP, blood pressure; eGFR, estimated glomerular filtration rate; ACR, albumin:creatinine ratio; CRP, C-reactive protein; ECR, Endotrophin:creatinine ratio.

### ECR correlates with eGFR, one-year relative change in eGFR and ACR

**Figure 2:**
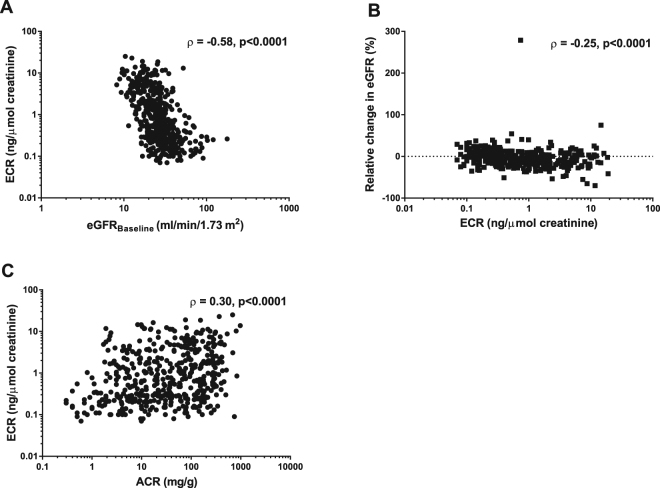
ECR correlates with eGFR, one-year relative change in eGFR and albuminuria. Correlation analysis between ECR and (**A**) baseline eGFR, (**B**) one-year relative change in eGFR, and (**C**) ACR are presented. Due to non-normal distribution of data, spearman’s rank correlation analysis was performed. For visualization, all values, except relative change in eGFR, were log-transformed. In each subfigure, spearman’s rho and significance level is shown.

As eGFR and ACR were respectively lower and higher in the higher ECR quartiles, we performed correlation analysis between ECR and eGFR, one-year relative change in eGFR and ACR (Fig. 2). As log-transformation did not lead to a Gaussian distribution of ECR, we performed Spearman rank correlation analysis. ECR had a moderate, inverse correlation with baseline eGFR (rho = −0.58, p < 0.0001, Fig. 2A). Out of the 499 patients, 381 (76.4%) had paired eGFR data available at 0 and 12 months and the one-year relative change in kidney function was calculated. ECR had an inverse, weak correlation with one-year relative change in eGFR (rho = −0.25, p < 0.0001; Fig. 2B) and a weak correlation with ACR (rho = 0.30, p < 0.0001; Fig. 2C).

### ECR is associated with one-year disease progression

**Figure 3:**
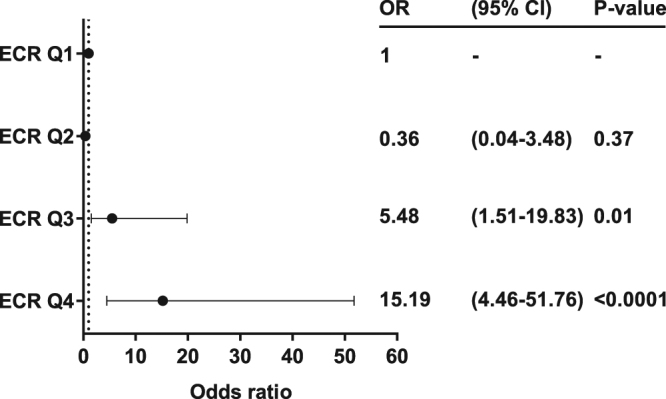
The association of ECR quartiles with one-year disease progression. Odds ratios with 95% CI were plotted for quartiles (Q) of ECR.

Forty six (11.1%) of 416 patients alive and with one year follow-up data had progressed. Relative to the lowest quartile, the third and fourth quartiles of ECR had odds ratios (ORs) for progression of 5.48 (Q3; 95% CI 1.51–19.83, p = 0.01), and 15.19 (Q4; 95% CI 4.46–51.76, p < 0.0001), respectively (Fig. 3).

Next we investigated whether the association of the third and fourth quartile of ECR with one-year disease progression was independent of traditional risk factors of disease progression, and thus adjusted for age, gender, eGFR and ACR^16^. Both Q3 and Q4 were independently associated with one-year disease progression with ORs of 3.68 (95% CI 1.06–12.83, p = 0.04) and 8.65 (95% CI 2.46–30.49, p = 0.001), respectively. The analysis was also performed with tertiles of ECR. Relative to the lowest tertile, the second and third tertile had ORs of 4.03 (95% CI 1.08–14.97, p = 0.04) and 17.19 (95% CI 5.13–57.60, p < 0.0001), respectively. Adjusted for age, gender, eGFR and ACR only the third tertile of ECR remained independently associated with one-year disease progression (OR 3.02, 95% CI 1.31–6.95, p = 0.009).

To investigate whether addition of ECR quartiles to the model of traditional risk factors, reported by Tangri *et al*.^16^, improved prediction of one-year disease progression, we performed category-free net reclassification index (cfNRI) and integrated discrimination improvement (IDI). The addition of ECR quartiles to the model increased prediction as seen by a significant increase in category-free net reclassification improvement (0.45, 95% CI 0.16–0.74, p = 0.002) and integrated discriminatory improvement (0.04, 95% CI 0.02–0.06, p < 0.001).

To further dissect the effect of other variables on the association of ECR with one-year disease progression, we adjusted ECR for eGFR and ACR alone and in combination, and for all variables that differed significantly between ECR quartiles including gender^16^ (Table 2). Adjustment of ECR for either eGFR or ACR did not markedly affect the association with one-year disease progression (all p < 0.001). Adjustment for eGFR and ACR in combination lead to a reduction in the association of ECR with one-year disease progression, but the association remained significant (per increase in 1 SD; OR 1.42, 95% CI 1.04–1.93, p = 0.03). Even after adjusting for all variables that differed significantly between ECR quartiles including gender^16^, the association remained significant (per increase in 1 SD; OR 1.63, 95% CI 1.15–2.32, p = 0.007).

**Table 2:** Association of ECR with one-year disease progression and development of ESRD.

Model	One-year disease progression		ESRD	
	OR^a^ (95% CI)	P-value	HR^a^ (95% CI)	P-value
Model a	1.55 (1.20–2.02)	0.0009	1.15 (1.00–1.31)	0.04
Model b	1.80 (1.37–2.35)	<0.0001	1.30 (1.16–1.00)	<0.0001
Model c	1.42 (1.04–1.93)	0.03	0.95 (0.81–1.11)	0.51
Model d	1.63 (1.15–2.32)	0.007	0.98 (0.83–1.16)	0.82

Data are odds ratio (OR) or hazard ratio (HR) with 95% CI as specified. One-year disease progression was defined as either a decline of eGFR of more than 30% or development of ESRD within one year. Urinary ECR was adjusted in four different models: Model a) ECR adjusted for eGFR; Model b) ECR adjusted for ACR; Model c) ECR adjusted for eGFR and ACR; and Model d) ECR adjusted for all variables with a univariable association with ECR (p < 0.1) and gender^16^. The variables included in the latter model (“Model d”) included age, gender, ethnicity, primary renal diagnosis, diabetes mellitus as comorbidity, age-adjusted CCI (score ≥ 5), PP, eGFR, and ACR. Due to missing data for some variables used for adjustment, the fully adjusted model only included 406 out of 416 patients (98%) with data available for one-year disease progression and 484 out of 499 patients (97%) for development of ESRD. Logistic regression analysis was used to analyze the association to one-year disease progression, and Cox proportional hazard regression analysis was used to analyze the association to development of ESRD. ECR, endotrophin:creatinine ratio; CCI, Charlson’s comorbidity index; PP, pulse pressure; eGFR, estimated glomerular filtration rate; ACR, albumin creatinine ratio. ^a^per increase in one standard deviation (1 SD) of ECR.

### Association of ECR with Development of ESRD

**Figure 4:**
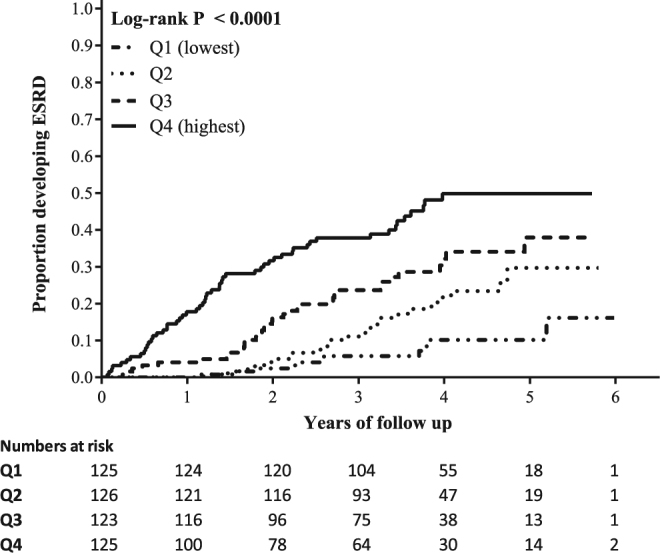
Cumulative Kaplan-Meier plot showing development of ESRD by ECR quartile. A table including the number of patients at risk by time has been inserted below the Kaplan-Meier curve. Log-rank significance is inserted in the figure. Q = quartiles.

One hundred and twenty-five (25.1%) of 499 patients developed ESRD during the follow-up period. ECR level when analysed as a continuous variable (Table 2) or by quartiles (Fig. 4) was significantly associated with the development of ESRD. Patients in Q2, Q3 and Q4 of ECR had hazard ratios (HR) of 2.41 (95% CI 1.19–4.90, p = 0.015), 3.92 (95% CI 1.99–7.73, p = 0.0001) and 7.20 (95% CI 3.76–13.78, p < 0.0001), respectively, relative to the lowest quartile. The number and percentage of patients advancing to ESRD in each ECR quartile were 11 (8.8%, Q1), 25 (19.8%, Q2), 35 (28.5%, Q3), and 54 (43.2%, Q4) (Log-rank χ^2^ = 55.3, p < 0.0001).

As was done for one-year disease progression, we investigated the effect of other variables on the association of ECR with development of ESRD. We adjusted ECR for eGFR and ACR alone and in combination, and for all variables that differed significantly between ECR quartiles, and gender^16^ (Table 2). Adjustment for eGFR markedly reduced the association of ECR with development of ESRD, but the association still remained significant (per increase in 1 SD; HR 1.15, 95% CI 1.00–1.31, p = 0.04). Adjustment for ACR revealed an independent association of ECR with development of ESRD (per increase in 1 SD; HR 1.30, 95% CI 1.16–1.00, p < 0.0001). When ECR was adjusted for eGFR and ACR in combination, the association with development of ESRD was lost. When ECR was adjusted for all variables that differed significantly between ECR quartiles and gender, ECR lost its association with the development of ESRD (per increase in 1 SD; HR 0.98, 95% CI 0.83–1.16, p = 0.82)(Table 2).

## Discussion

In the present study we tested nonfibrotic and fibrotic kidney biopsies for the presence of endotrophin, and measured levels of ECR in samples from patients with high-risk CKD. To our knowledge, this is the first study that studies the presence of endotrophin in biopsies, and investigates the prognostic potential of ECR in CKD. The main finding was that ECR was associated with one-year disease progression independent of traditional risk factors. We also show that addition of ECR quartiles to a model of traditional risk factors significantly increased discrimination of events and nonevents for one-year disease progression. Our findings also indicate that endotrophin is mainly found in the interstitial matrix in fibrotic kidneys, and localizes to areas of high COL VI expression. Together our data indicate that endotrophin is produced in fibrotic kidneys and that ECR may reflect disease activity in CKD patients.

Renal fibrosis is a hallmark of CKD progression^1,3^. Collagen is the major constituent of fibrotic lesions and consists of multiple types. The major focus to date on collagen in fibrosis has been on collagen type I and type III^4^. However, there is now an increasing interest in the role of COL VI across different organs. Endotrophin is generated by the cleavage of the alpha-3 chain of COL VI^11,12^. Due to sequence overlap in endotrophin and the pro-peptide of COL VI, endotrophin may be a potential surrogate biomarker for COL VI formation. Endotrophin is a matrikine with multiple pleiotropic effects including the potential to amplify fibrotic processes^13^. However, there have been no studies to date investigating the presence of endotrophin in fibrotic kidneys and the association of urinary levels of endotrophin with disease progression in CKD patients.

In normal kidney COL VI co-localised to areas with collagen matrix, as identified by Masson’s trichome, in a distribution consistent with that previously described^17^. Endotrophin is not a component of the steady-state COL VI molecule but is released during deposition in the extracellular matrix^11^. Consistent with this there was no detectable endotrophin by immunohistochemistry in histologically normal kidney, indicating that any new production of endotrophin in this setting was below the detection threshold for the antibody. However, in established fibrosis there was considerable expression of COL VI and this co-localised with prominent expression of endotrophin. This suggests that COL VI is actively produced in the fibrotic foci and thus the biologically active matrikine endotrophin is also produced.

We utilised a prospective cohort of patients with progressive CKD to explore the association of ECR with one-year disease progression and development of ESRD. We found a significant negative correlation between urinary ECR and kidney function and between ECR and one-year relative change in kidney function. Urinary ECR levels were independently associated with an increased risk of one-year disease progression, and the third and fourth quartiles of ECR remained independently associated with the rate of progression after adjustment for the traditional risk factors age, gender, eGFR and ACR^16^. ECR as a continuous variable also remained significantly associated with one-year disease progression, even when fully adjusted for potential confounders, including the traditional risk factors^16^.

When looking at the univariable association between ECR and development of ESRD a striking prognostic ability was observed, even after adjustment of ECR by eGFR and ACR alone. The adjustment for eGFR reduced the risk, but the association of ECR to development of ESRD was still significant indicating that there is an association independent of kidney function. However, when adjusted for eGFR and ACR in combination and adjusted for all potential confounders and gender, ECR lost its ability to predict development of ESRD. An explanation for the marked impact of eGFR on ECR could be that ECR is a measure of COL VI formation, and therefore may reflect renal fibrosis. As renal fibrosis drives the loss of kidney function^1,3^, eGFR may be part of the pathway which is driven by endotrophin and the exacerbated COL VI deposition. Adjusting for eGFR might be adjusting for the same pathway as endotrophin thus attenuating the association of ECR with development of ESRD. It is therefore interesting that ECR is independently associated with one-year disease progression, as this might indicate that ECR is associated with disease activity and thus rate of progression. In the cohort, all patients were designated as having high-risk CKD to ensure that there was a substantial amount of patients with a rapid disease progression. All patients therefore had advanced CKD, and thus a relatively low eGFR. A future study testing the prognostic ability of ECR in a cohort of patients with mild CKD should be conducted to verify whether ECR is associated with disease progression independent of kidney function.

An interesting finding was that albuminuria, as assessed by ACR, did not affect the association of ECR with either one-year disease progression or development of ESRD. The presence of endotrophin in urine is therefore not likely to be solely due to an increased filtration of proteins into the urine through the glomerular basement membrane, and might reflect a release of endotrophin from the fibrotic renal extracellular matrix.

Patients with a primary renal diagnosis of diabetes presented higher ECR levels. A link between type 2 diabetes mellitus and endotrophin was previously established by our group^18^. In this study, levels of serum endotrophin predicted which patients could benefit from insulin-sensitising treatment as assessed by lowering of blood glucose^18^. This study suggested that endotrophin may be able to isolate a subgroup of diabetic patients with high disease activity. As diabetes is the main cause of CKD, and COL VI is upregulated in kidneys of diabetic patients^8^, our findings add to the hypothesis that high endotrophin levels reflects disease activity favouring progression of disease.

Patients with glomerulonephritis had lower levels of ECR than diabetic patients. However, patients with diabetes as a primary renal diagnosis had a lower eGFR than patients with glomerulonephritis (data not shown). As we showed a negative correlation between eGFR and ECR levels, the discrepancy might be explained by a more advanced disease stage of the diabetic patients, than those with glomerulonephritis in the cohort.

Whilst we identify an association between endotrophin and renal outcomes, it is not known whether endotrophin is causally linked to progression of renal disease. Also, even though COL VI is upregulated in pathologies affecting the kidneys^8,9^, endotrophin may also increase in comorbidities present in many patients with kidney disease. In animal studies, COL VI was upregulated in diabetic rats with hypertension and cardiac fibrosis^19^ and in rats with myocardial infarction^20^. As mice with COL VI alpha-1 chain deficiency had less cardiac damage following myocardial infarction compared to controls, this might suggest that COL VI is involved in an abnormal wound-healing^21^. The same trend was seen in humans where COL VI was upregulated in atherosclerotic lesions^22^, and in hypertrophic cardiomyopathy where COL VI levels correlated with cardiac dysfunction^23^. One possible explanation for the hazardous nature of COL VI was seen in *in vitro* experiments where plating of cardiac fibroblasts on COL VI induced myofibroblast differentiation^20^. This might also be seen *in vivo*, where COL VI was upregulated and coincided with increased myofibroblast content in a model of myocardial infarction in rats^20^. As type I and III collagen increase proliferation of cardiac fibroblasts *in vitro*, the combined increase of type I, III, and VI collagen in fibrosis could form a matrix which sustains a vicious fibrotic cycle through proliferation and differentiation of fibroblasts^20^. Overall the findings suggest that COL VI is not only a consequence of fibrotic events, but that its presence and post-translational modifications could also drive pathology through the release of endotrophin^24^.

We have previously shown that high levels of serum endotrophin were independently associated with mortality^14^. In the current study there was no association with mortality for ECR as a continuous variable or as quartiles, and the study only focused on endpoints related to the kidneys (i.e. one-year disease progression and ESRD). The analysis on serum endotrophin did not focus on one-year disease progression^14^, but post-hoc analysis showed that the fourth quartile of serum endotrophin was lowly, but significantly associated with one-year disease progression after adjustment for the same traditional risk factors that were used in the current study. However, the association of ECR with one-year disease progression in the current study was more prominent and both the third and the fourth quartiles of ECR were independently associated with it. This may indicate that endotrophin in urine reflects the pathological changes taking place in the kidney, whereas the serum levels are influenced by systemic changes due to the disease. This could also explain the marked difference when analysing serum and urine levels of endotrophin in respect to all-cause mortality.

The strengths of this study are that endotrophin was shown *in situ* in human renal fibrosis and was evaluated in a large prospective cohort of high-risk CKD that incorporates multiple risk factors for the clinical end-points enabling robust analyses.

The limitations of the study include it being a single-centre cohort, and our reporting of an association of endotrophin and clinical outcomes with no supporting mechanistic data. However this study does identify ECR as a strong candidate molecule for evaluation in a stratified medicine model of CKD progression. There is an urgent need for the development of clinical trial models where early changes in pathology in response to therapy can stratify patients for clinical benefit. We propose that endotrophin should be evaluated as a candidate molecule for this role in CKD. This is supported by our findings, where the highest two quartiles of ECR were independently associated with one-year disease progression, irrespective of traditional risk factors including age, gender, eGFR and ACR. As fast progressors are believed to be more likely to respond to therapy, stratification by ECR is highly interesting as it, due to sequence overlap with the pro-peptide, might reflect both COL VI formation and levels of matrikine with potential deleterious effects.

In conclusion, endotrophin is present *in situ* in human renal fibrosis at sites of COL VI deposition and ECR is independently associated with one-year disease progression in CKD patients.

## Methods

### Human renal tissue

Previous studies have shown that COL VI is upregulated in fibrosis of the kidney^8,9^. To assess the expression of endotrophin in relationship to COL VI we studied *in situ* expression in human kidney disease. Human renal tissue was obtained from nephrectomized specimens with renal fibrosis from five patients with advanced hydronephrosis and/or chronic pyelonephritis as previously described^25^. Control kidney tissue comprised histologically (uninvolved) normal cortex from nephrectomy specimens of five patients with renal cell carcinoma or trauma. All human samples were analysed in a retrospective anonymised manner, after the approval of the local ethics committee on human research in accordance with the Helsinki Declaration of 1975, as revised in 2000 (No. EK244-14).

### Assessment of renal tissue

#### Tissue preparation

Tissue for light microscopy and immunohistochemistry was fixed in methyl Carnoy’s solution and embedded in paraffin and 1 mm sections were processed as previously described^26^.

#### Masson’s trichrome staining

Sections of human kidney tissue were deparaffinized and rehydrated through absolute ethanol, 96% ethanol, 70% ethanol, and finally distilled water. Sections were stained by incubation in Weigert’s iron hematoxylin solution for 10 minutes, followed by running warm tap water for 10 minutes, and finally rinsed in distilled water. Sections were incubated in Biebrich scarlet-acid fuchsin solution for 10–15 minutes, and subsequently washed in distilled water. The sections were incubated in phophomolybdic-phosphotungstic acid solution for 10–15 minutes and transferred directly to aniline blue solution and stained for 5–10 minutes. Following a brief rinse in distilled water, sections were differentiated in 1% acetic acid for 2–5 minutes, washed in distilled water, and quickly dehydrated through 96% ethanol, absolute ethanol, and finally toluene. Sections were mounted with resinous mounting medium.

#### Immunohistochemistry

Human kidney tissue were deparaffinized in toluene, rinsed in absolute ethanol and blocked with 1.05% H_2_O_2_ in absolute ethanol for 20 minutes. Slides were rehydrated by incubation in 96% ethanol, 70% ethanol, and finally tap water. The slides were incubated in citrate buffer pH 6.0 overnight at 60 °C to unmask epitopes. After incubation the slides were cooled to room temperature and washed in Tris-buffered saline (TBS). To prevent non-specific background staining, slides were blocked by incubation with 0.5% casein in TBS. Antibody was diluted in TBS as specified: αCOL VI (final concentration 2.0 µg/ml, abcam-6588, abcam), α-endotrophin (final concentration 2.7 µg/ml, PRO-C6, Nordic Bioscience), irrelevant isotype matched IgG antibody (final concentration 2.0 µg/ml, Code X0931, DAKO), and 200 µL was added to each slide followed by overnight incubation at 4 °C. The slides were washed in TBS and incubated with the EnVision^TM^ system HRP kit according to the manufacturer’s instructions (Dako). A secondary peroxidase labeled polymer anti-mouse was added for 30 minutes, followed by two washing steps in TBS. The tissue was covered with Super Enhancer^TM^ followed by two washing steps in TBS. 3.3′-diaminobenzidine (DAP+) substrate chromatogen solution (0.1 nM chromogen in substrate buffer) was added and incubated for 8 minutes at room temperature. The colorimetric reaction was stopped by washing in distilled water. The tissue was briefly counterstained with Meyer’s hematoxylin and rinsed in tap water. The slides were dehydrated in 96% ethanol, absolute ethanol, and finally in toluene. As a negative control, irrelevant isotype matched IgG antibody was used (Code X0931, DAKO) in place of the primary antibody; no unspecific staining was observed.

### Study subjects

#### Study description

Four hundred and ninety-nine participants from the Renal Impairment in Secondary Care (RIISC) Study (NCT01722383) were included in the study. Detailed methodology of the RIISC study has previously been described^27^. The cohort was designed as a prospective observational cohort study designed to identify determinants of adverse outcomes in CKD.

In brief, patients in nephrology clinics in a single renal centre in Birmingham, UK, with non-dialysis high-risk CKD, were invited to participate in the study. High-risk CKD was as defined by the UK National Institute for Health and Care Excellence 2008 CKD guideline, and comprised one or more of: eGFR <30 mL/min/1.73 m^2^, or eGFR 30–59 mL/min/1.73 m^2^ with a decline of ≥5 mL/min/1.73 m^2^/year or ≥10 mL/min/1.73 m^2^/5 years, or a urinary ACR ≥70 mg/mmol on three occasions^28^. Patients were excluded if they had received immunosuppression or had started renal replacement therapy (RRT). Patients consented to follow-up for 10 years, or either initiation of RRT or death. Ethical approval was granted by South Birmingham Local Research Ethics Committee (reference: 10/H1207/6). All patients provided written informed consent, and the study was conducted in accordance with the Declaration of Helsinki.

All patients received standard of care during the study. The visits occurred between April 2011 and September 2014. The last outcome data collection took place on 31^st^ of January 2017 at which point patients, who had not reached a study end-point, were censored. Median follow-up time, calculated using the reverse Kaplan-Meier method, was 46 months (range 0–72).

#### Demographic and clinical data

All participants had demographic, clinical, and laboratory data collected at recruitment and during follow-up. Comorbidities were recorded and age-adjusted Charlson Comorbidity Index (CCI) of each individual was calculated^29^. Socioeconomic status was assessed using the Index of Multiple Deprivation (IMD 2010); lower scores and ranks indicate greater deprivation^30^. Renal diagnosis was set as ‘Other/Unknown’, when CKD was due to uncertain aetiology (33.3%), or if it had not been collected (8.6%).

#### Blood pressure

Blood pressure (BP) was measured using the BpTRU fully automated sphygmomanometer (BpTRU Medical Devices, Coquitlam, BC, Canada), which is obtained by an average of six BP readings at one-minute intervals following a five-minute rest period, and is comparable to mean daytime BP from 24-hour ambulatory BP monitoring^31^. Mean arterial pressure (MAP) was calculated with mean systolic blood pressure (SBP) and mean diastolic blood pressure (DBP), with the equation: DBP + ((SBP − DBP)/3). Pulse pressure (PP) was calculated as the difference between SBP and DBP.

### Laboratory Analyses

#### Processing of samples

Urine samples were processed immediately after collection according to pre-defined standard operating procedures and stored at −80 °C until analysis. Biochemistry results from the local clinical laboratory were obtained from tests performed in accordance with the current standard of care.

#### Endotrophin

Urinary endotrophin level was measured using a competitive enzyme-linked immunosorbent assay (PRO-C6) developed by Nordic Bioscience, Denmark^32^. The monoclonal antibody employed in the ELISA specifically detects the last 10 amino acids of the alpha-3 chain of COL VI (_3168′_KPGVISVMGT_3177′_)^32^. Intra- and interassay variation of the assay were below 3.2 and 7.9%, respectively. To account for variations in urine concentration, urinary endotrophin was divided by urinary creatinine, and the endotrophin:creatinine ratio (ECR) used in analyses.

#### Creatinine, urinary ACR, and C-reactive protein

Serum creatinine measurements were performed on a Roche Modular Analyser using a blank rated compensated Jaffe reaction, and eGFR was estimated using the creatinine-based CKD-EPI equation^33^. Urinary creatinine was measured using the ADVIA 1800 Chemistry System (Bayer HealthCare). Urinary ACR was measured using a Roche Hitachi 702 analyser. C-reactive protein (CRP) was measured using the Full Range C-Reactive Protein Kit on a SPA^TM^ automated PLUS turbidimeter (The Binding Site Group Ltd, UK). The normal range for CRP is between 0.1 and 9 mg/L, with 90 percent below 3 mg/L^34^.

### Statistical analyses

Baseline characteristics are described as frequency and percentage for categorical variables, and as median and interquartile range (IQR) for continuous variables. As there was little missing data, complete case analysis was performed, unless otherwise stated.

The primary outcomes comprised: 1) one-year disease progression of chronic kidney disease as defined by a decline in eGFR of ≥30% or development of ESRD within 12 months and 2) development of ESRD. Development of ESRD was defined as initiation of RRT by either dialysis or renal transplantation and was captured through the use of a local database of all patients who initiated RRT. The composite endpoint for one-year disease progression was based on a study by Coresh *et al*. stating that a ≥30% decline in eGFR is associated with a major increased relative risk of progressing to ESRD^35^. To not exclude fast progressing patients from the analysis, we included patients that developed ESRD during the first one-year of follow-up in the composite endpoint.

Urinary endotrophin:creatinine ratio (ECR) was analysed per increase in one standard deviation (1 SD) and as a categorical variable by dividing the cohort into quartiles (Q1-4). As transformation of ECR did not lead to a Gaussian distribution (assessed by a D’Agostino-Pearson omnibus normality test), non-parametric analysis were conducted when possible.

Differences between ECR quartiles in respect to baseline characteristics were assessed using a Chi-squared test for categorical variables, or the non-parametric Kruskal-Wallis test for continuous variables.

To assess linear association, correlations between ECR and eGFR, one-year relative change in eGFR, and ACR were carried out by the non-parametric Spearman’s rank correlation. One-year relative change in eGFR was calculated as the difference between baseline eGFR and eGFR at one year divided by baseline eGFR.

Univariable and multivariable logistic regression analyses were used to analyse the association of urinary ECR with one-year disease progression and Cox proportional hazards regression analyses was used to analyse the association with development of ESRD. Association of ECR quartiles with one-year disease progression was analysed with logistic regression analysis. Associations were adjusted for the main variables used to predict risk of disease progression in the clinic namely age, gender, eGFR, and ACR^16^. To further assess whether other commonly obtained patient characteristics affected the association of ECR with outcomes, we adjusted ECR for eGFR (Model a), ACR (Model b), eGFR and ACR (Model c), and all variables with a univariable association with ECR quartiles (p < 0.1), including gender^16^ (Model d). Due to missing data for some variables used for adjustment, the fully adjusted model only included 406 out of 416 patients (98%) with data available for one-year disease progression and 484 out of 499 patients (97%) for development of ESRD.

When variables were highly correlated, only the one with the highest association between ECR quartiles was included as a candidate variable in model adjustment. As an example, pulse pressure (PP) was selected for one-year disease progression and development of ESRD.

Kaplan-Meier survival analysis was performed for quartiles of ECR against development of ESRD, and significant differences between quartiles was assessed by a Log-rank (Mantel-Cox) test.

All two-tailed p-values below 0.05 were considered significant. Statistical analyses were performed using MedCalc (Ostend, Belgium), R (version 3.2.4), or GraphPad Prism (version 7.00).

### Net reclassification improvement (NRI) and integrated discrimination improvement (IDI)

The cfNRI and IDI have been increasingly applied to determine the added value of a biomarker to an existing test or model^36–39^. We used these measures to determine whether the addition of ECR quartiles added incremental predictive value to a model containing traditional risk factors for disease progression (age, gender, eGFR, and ACR)^16^. The cfNRI and IDI were calculated in R with the package PredictABEL (version 1.2–2).

It can be interpreted as a measurement of an increase in event rate among those reclassified as having higher risk and decrease in event rate among those reclassified as having lower risk.

The cfNRI was calculated as follows. For patients with events, reclassification was the difference between the fraction of patients who were reclassified as being at higher risk and the fraction of patients who were reclassified to having lower risk:

1 $${{\rm{Fraction}}}_{{\rm{event}}-{\rm{i}}}=\frac{{\rm{number}}\,\,{\rm{of}}\,\,{\rm{events}}\,\,{\rm{with}}\,\,{\rm{increased}}\,\,{\rm{predicted}}\,\,{\rm{risk}}\,\,{\rm{after}}\,\,{\rm{addition}}\,\,{\rm{of}}\,\,{\rm{ECR}}}{{\rm{number}}\,\,{\rm{of}}\,\,{\rm{events}}}$$

2 $${{\rm{Fraction}}}_{{\rm{event}}-{\rm{d}}}=\frac{{\rm{number}}\,\,{\rm{of}}\,\,{\rm{events}}\,\,{\rm{with}}\,\,{\rm{decreased}}\,\,{\rm{predicted}}\,\,{\rm{risk}}\,\,{\rm{after}}\,\,{\rm{addition}}\,\,{\rm{of}}\,\,{\rm{ECR}}}{{\rm{number}}\,\,{\rm{of}}\,\,{\rm{events}}}$$

3 $${{\rm{cfNRI}}}_{{\rm{event}}}={{\rm{Fraction}}}_{{\rm{event}}-{\rm{i}}}-{{\rm{Fraction}}}_{{\rm{event}}-{\rm{d}}}$$

For patients without events (nonevent), reclassification was the difference between the fraction of patients who were reclassified to lower risk and the fraction of patients who were reclassified to higher risk:

4 $${{\rm{Fraction}}}_{{\rm{nonevent}}-{\rm{d}}}=\frac{{\rm{number}}\,\,{\rm{of}}\,\,{\rm{nonevents}}\,\,{\rm{with}}\,\,{\rm{decreased}}\,\,{\rm{predicted}}\,\,{\rm{risk}}\,\,{\rm{after}}\,\,{\rm{addition}}\,\,{\rm{of}}\,\,{\rm{ECR}}}{{\rm{number}}\,\,{\rm{of}}\,\,{\rm{nonevents}}}$$

5 $${{\rm{Fraction}}}_{{\rm{nonevent}}-{\rm{i}}}=\frac{{\rm{number}}\,\,{\rm{of}}\,\,{\rm{nonevents}}\,\,{\rm{with}}\,\,{\rm{increased}}\,\,{\rm{predicted}}\,\,{\rm{risk}}\,\,{\rm{after}}\,\,{\rm{addition}}\,\,{\rm{of}}\,\,{\rm{ECR}}}{{\rm{number}}\,\,{\rm{of}}\,\,{\rm{nonevents}}}$$

6 $${{\rm{cfNRI}}}_{{\rm{nonevent}}}={{\rm{Fraction}}}_{{\rm{nonevent}}-{\rm{d}}}-{{\rm{Fraction}}}_{{\rm{nonevent}}-{\rm{i}}}$$

The total cfNRI is the sum of correct reclassification among patients with (equation (3)) and without (equation (6)) event:

7 $${{\rm{cfNRI}}}_{{\rm{total}}}={{\rm{cfNRI}}}_{{\rm{event}}}+{{\rm{cfNRI}}}_{{\rm{nonevent}}}$$

The maximum total cfNRI is 200%, indicating that the predicted risks for all subjects with events are increased, and all subjects without events are decreased.

The IDI was calculated as follows.

8 $${{\rm{IDI}}}_{\mathrm{event}+\mathrm{ECR}}=\frac{{\rm{sum}}\,\,{\rm{of}}\,\,{\rm{probability}}\,\,{\rm{of}}\,\,{\rm{event}}\,\,{\rm{after}}\,\,{\rm{addition}}\,\,{\rm{of}}\,\,{\rm{ECR}}}{{\rm{number}}\,\,{\rm{of}}\,\,{\rm{events}}}$$

9 $${{\rm{IDI}}}_{{\rm{event}}-{\rm{ECR}}}=\frac{{\rm{sum}}\,\,{\rm{of}}\,\,{\rm{probability}}\,\,{\rm{of}}\,\,{\rm{event}}\,\,{\rm{without}}\,\,{\rm{addition}}\,\,{\rm{of}}\,\,{\rm{ECR}}}{{\rm{number}}\,\,{\rm{of}}\,\,{\rm{events}}}$$

10 $${{\rm{IDI}}}_{{\rm{event}}}={{\rm{IDI}}}_{{\rm{event}}+{\rm{ECR}}}-{{\rm{IDI}}}_{{\rm{event}}-{\rm{ECR}}}$$

11 $${{\rm{IDI}}}_{{\rm{nonevent}}-{\rm{ECR}}}=\frac{{\rm{sum}}\,\,{\rm{of}}\,\,{\rm{probability}}\,\,{\rm{of}}\,\,{\rm{event}}\,\,{\rm{without}}\,\,{\rm{addition}}\,\,{\rm{of}}\,\,{\rm{ECR}}}{{\rm{number}}\,\,{\rm{of}}\,\,{\rm{nonevents}}}$$

12 $${{\rm{IDI}}}_{{\rm{nonevent}}+{\rm{ECR}}}=\frac{{\rm{sum}}\,\,{\rm{of}}\,\,{\rm{probability}}\,\,{\rm{of}}\,\,{\rm{event}}\,\,{\rm{after}}\,\,\,{\rm{addition}}\,\,{\rm{of}}\,\,{\rm{ECR}}}{{\rm{number}}\,\,{\rm{of}}\,\,{\rm{nonevents}}}$$

13 $${{\rm{IDI}}}_{{\rm{nonevent}}}={{\rm{IDI}}}_{{\rm{nonevent}}-{\rm{ECR}}}-{{\rm{IDI}}}_{{\rm{nonevent}}+{\rm{ECR}}}$$

The IDI is the sum of correct reclassification among patients with (equation (10)) and without (equation (13)) event:

14 $${\rm{IDI}}={{\rm{IDI}}}_{{\rm{event}}}+{{\rm{IDI}}}_{{\rm{nonevent}}}$$

### Data availability

The datasets generated and analysed during the current study are available from the corresponding author on reasonable request.
